# Rapid Localization and Mapping Method Based on Adaptive Particle Filters †

**DOI:** 10.3390/s22239439

**Published:** 2022-12-02

**Authors:** Anas Charroud, Karim El Moutaouakil, Ali Yahyaouy, Uche Onyekpe, Vasile Palade, Md Nazmul Huda

**Affiliations:** 1 Laboratory of Engineering Sciences, Multidisciplinary Faculty of Taza, Sidi Mohamed Ben Abdellah University, Taza 35000, Morocco; 2 Computer Science, Signals, Automatics and Cognitivism Laboratory, Sciences Faculty of Dhar El Mahraz, Sidi Mohamed Ben Abdellah University, Fès-Atlas 30000, Morocco; 3 School of Science, Technology and Health, York St John University, York YO31 7EX, UK; 4 Centre for Computational Science and Mathematical Modelling, Coventry University, Priory Road, Coventry CV1 5FB, UK; 5 Department of Electronic and Electrical Engineering, Brunel University London, Kingston Ln, Uxbridge UB8 3PH, UK

**Keywords:** autonomous driving, feature extraction, mapping, localization, self-driving vehicles, SLAM

## Abstract

With the development of autonomous vehicles, localization and mapping technologies have become crucial to equip the vehicle with the appropriate knowledge for its operation. In this paper, we extend our previous work by prepossessing a localization and mapping architecture for autonomous vehicles that do not rely on GPS, particularly in environments such as tunnels, under bridges, urban canyons, and dense tree canopies. The proposed approach is of two parts. Firstly, a K-means algorithm is employed to extract features from LiDAR scenes to create a local map of each scan. Then, we concatenate the local maps to create a global map of the environment and facilitate data association between frames. Secondly, the main localization task is performed by an adaptive particle filter that works in four steps: (a) generation of particles around an initial state (provided by the GPS); (b) updating the particle positions by providing the motion (translation and rotation) of the vehicle using an inertial measurement device; (c) selection of the best candidate particles by observing at each timestamp the match rate (also called particle weight) of the local map (with the real-time distances to the objects) and the distances of the particles to the corresponding chunks of the global map; (d) averaging the selected particles to derive the estimated position, and, finally, using a resampling method on the particles to ensure the reliability of the position estimation. The performance of the newly proposed technique is investigated on different sequences of the Kitti and Pandaset raw data with different environmental setups, weather conditions, and seasonal changes. The obtained results validate the performance of the proposed approach in terms of speed and representativeness of the feature extraction for real-time localization in comparison with other state-of-the-art methods.

## 1. Introduction

The recent advances in the development of Autonomous Vehicles (AVs) offer great potential for improving road safety. AVs offer a market-ready solution to reduce road-related crashes and fatalities [1]. Achieving these goals requires an effective autonomous vehicle localization system that can locate the vehicle in its environment. The combination of positioning and orientation techniques for localization and appropriate mapping methods are required for trajectory planning and safe navigation of AVs [2].

The classical approach for locating AVs is to use a Global Navigation Satellite System (GNSS). Triangulation of at least three GNSS signals provides information for locating AVs on the road. However, GNSS reliability is compromised by visibility problems in tunnels, canyons, under bridges, and dense tree canopies, as well as by multipath reflections and signal delay [3,4,5]. A common approach employed during the absence of GNSS signals is to use the Inertial Navigation Systems (INS), which can provide continuous position and attitude information based on the vehicle’s acceleration and heading speed provided by the accelerometer and the gyroscope, respectively [5]. However, the accuracy of the INS deteriorates exponentially during the double integration of the acceleration measurements to position and of the azimuthal velocity measurements to azimuth [6]. Similar to the accelerometers of the INS, wheel odometry has been investigated as a solution for position determination in GNSS-deprived environments [7,8]. By using measurements from wheel encoders (which measure the wheel speed of the vehicle) and integrating the linear velocity of the vehicle derived from the wheel speed, it is possible to determine the position of the vehicle with one less integration step compared to the accelerometer measurement [8]. However, like the INS, wheel encoder measurements suffer from errors that are exponentially amplified when integrated into position [8]. To provide a more accurate position and orientation estimation, needed for a safer and more effective navigation solution in GNSS-deprived environments, several machine learning methods based on recurrent neural networks [5,6,7,8,9,10,11,12,13] have been proposed to learn the errors present in the wheel encoder, accelerometer, and gyroscope measurements. However, Black Box type of models, as usually encountered in deep learning, can pose several challenges, as they require large amounts of data to be properly trained in order to provide reliable prediction results, in addition to their decisions not being transparent to human users. Particle Filters (PF) have also been shown to be capable of accurately modelling measurement errors in similar types of applications. PFs have several advantages that can be used for localization. For example, PFs can handle nonlinear trajectories [14], can track multiple objects varying in time [15], and can handle occlusion and overlap [15]. Moreover, the PFs approach does not require prior assumptions, such as a Gaussian distribution of the data, such as the Kalman filter [16]. In its early days, PF was used in signal processing to estimate states based on observed variables [17]. Since then, PF has been adapted to solve localization problems [18,19,20,21]. In practice, PF (also called sequential Monte Carlo) uses the Markov assumption based on a concept that relates the state (in this case, the position) $X_{t}$ to the previous state $X_{t-1}$. The algorithm begins by generating thousands of particles representing candidate positions. A weight is assigned to each particle. The higher the weight’s value, the higher the probability that the particle is in the vehicle’s position. PFs offer the opportunity to explore the advantages of data from multiple sources.

Nevertheless, the above-described methods are not capable of providing lane-level accuracy using the information provided only by the INS and/or wheel encoder sensors. Camera and Light Detection and Ranging (LiDAR) are sensors commonly combined with the INS to provide lane-level localization. They are widely used in intelligent vehicles to provide an accurate representation of the vehicle’s environment, i.e., the objects around the vehicle and the distances between them. A technique called ”registration” [1] can be used to locate vehicles by aligning LiDAR scans and images from the camera. Typically, this method tracks the movement between keyframes, producing a transformation matrix that gives a translation vector *T* and a rotation matrix *R* from the first keyframe to the second keyframe [22]. Thus, the position of the vehicle follows the transformation described below in Equation (1).

(1) $$X_{t}=R*X_{t-1}+T$$

where $X_{t}$ is the position of the vehicle at time t, and $X_{t-1}$ is the position of the vehicle at time t−1.

The Iterative Closest Point (ICP) is one method that applies this registration concept [1,23]. It matches LiDAR scans by minimizing the cost function.

(2) $$arg\;\underset{R,T}{min}\left\{\frac{1}{M}\sum_{j=1}^{M}{∥a_{j}-\left(Rb_{j}+T\right)∥}_{2}\right\}\times$$

where ${\left\{a_{j}\right\}}_{j},{\left\{b_{j}\right\}}_{j}$ are the 3D coordinates points of the two lidar scans *A* and *B*, respectively, and *M* is the number of points inside *A* (or *B*). The idea is to find a matrix of translations and rotations that accurately represents the similarities between these two scans. Thus, the vehicle’s motion will also follow the same transformation as shown in Equation (1) [24]. However, due to measurement errors, the ICP algorithm is not able to provide good accuracy, especially in the long-term matching of nonlinear trajectories [25]. Therefore, the authors of [26] proposed the Normal Distribution Transform (NDT), which represents each group of 3D points included in a voxel (a 3D cube) by a probability distribution. The advantage of this representation is its ability to provide better results despite measurement errors and difficulties in detecting similarities between scans. However, these ‘registration’ methods are usually computationally time-consuming and do not work well when the number of feature points matched is not sufficient. [27] which complicates the process of finding the optimal parameters R and T needed to minimize the cost function in Equation (2). Developing a highly accurate and real-time efficient registration method is still an active research area in autonomous driving [27].

In this study, we propose a fuzzy K-means clustering technique to represent the environment efficiently, which facilitates the process of finding similar parts between the scan of the local map t and the corresponding parts on the global map. The global map contains information about the object in the trajectory. After generating these maps (local and global), an adaptive particle filter is used to localize the vehicle along the trajectory. The Adaptive Particle Filter (APF) is a particle filtering method supported by a resampling technique that allows the particles to follow approximately the actual positions of the trajectory and maintain accuracy. The APF uses the created local and global map to distinguish the best candidate particles for position calculation.

In this paper, we extend the work conducted in [28] on non-semantic feature map reduction using K-means, global map generation using GMM, and localization using particle filters by adding a resampling technique to the particle filter (i.e., employing the adaptive filter) to obtain a more accurate localization solution. In addition, we further investigated different parameter settings of the particle filter and the effect of the resampling extension on the performance of our proposed localization and mapping technique.

## 2. Related Work

Three approaches to fuse information from different sensors were found to be the most popular. These include parametric filters, such as extended Kalman filters; nonparametric filters, such as particle filters; and finally, least squares approaches, such as beam adaptation and graph-based Simultaneous Localization and Mapping (SLAM). In general, these methods use measurements from motion sensors to track vehicle motion, i.e., translation and rotation between two consecutive keyframes, and explore measurement sensors, i.e., LiDAR scans or images, as criteria to accurately approximate positions. Map features represent objects from the vehicle’s environment, or at least part of them, by their coordinates, which reduce the calculation time of the matching process and provide a clear representation of the map. There are two types of features: semantic and non-semantic features. Semantic features such as poles, trees, buildings, and sidewalks are widely used in feature mapping and vehicle tracking. The authors of [18] detected poles as feature landmarks by converting point clouds provided by the LiDARs into voxels, and the cells with an acceptable number of point clouds were connected vertically with a similar voxel. Each candidate pole has a certain number of connected cells (vertical sense). The landmarks were also fitted with cylinders to ensure reliability and extract pole parameters. A particle filter was used to perform the localization process. The method showed good accuracy on the Kitti dataset. Better localization accuracy was also achieved by poles and wall detection by Kummerl et al. [19]. In [20], the particle filter was used to localize the vehicle based on an algorithm that detects poles based on the intensity of the group of points in a voxel grid map. The algorithm was found to be able to detect landmarks such as trees, streetlights, and telegraph poles and determine their boundaries. Moreover, it detects the starting and ending points of the poles. A probabilistic grid map was implemented in [21] to identify voxels with high beta probability distribution values. The authors investigated the idea of isolation (i.e., poles are usually isolated from the surrounding space) and proposed a mathematical formulation that assigns a score to a pole candidate based on the intensity values inside and outside the pole. The closer the value is to 1, the more likely it is a pole. Their approach also used a particle filter to perform the localization task and compared its performance to other published methods. An accumulative positioning error of 7.67 m was registered in the University of Michigan North Campus Long-Term vision (NCLT) dataset [29] over 147.3 km of trajectory. Moreover, 0.096 m of the positioning error was registered in sequence 0009 of the Kitti dataset [30]. In article [31], the same probability maps were also used to identify walls and buildings in the vicinity. A Kalman filter was then applied to locate vehicles using the author’s dataset. In general, the integration of such semantic feature-based methods is computationally intensive. Moreso, these methods do not work efficiently in all environments, i.e., the environment must have texture and contain the desired features. Based on clustering methods, our proposed approach detects non-semantic features which exist in any environment, even with fewer objects’ textures.

Recent works have used this approach, such as the family of Oriented fast and Rotating Beams—SLAM (ORB-SLAM) [32,33,34], which extracted the camera position in each frame by matching the ORB features between keyframes based on local bundle adjustment, and the ORB features were used to generate maps of the environment. A tracking step was proposed to assist the system in re-locating, location awareness, or matching frames. These feature points are stored in a robust DataBase (DB) architecture called DBoW2 [35]. The ORB-SLAM system was tested on real data and has achieved high accuracy in point tracking, contour locking, and frame localization. However, weather fluctuations and insufficient brightness are the main problems with camera-based methods [1]. Hungar et al. [36] used DAISY [37] descriptors to illustrate 2D reflection maps of aligned point clouds. The remaining maps were fed an intensity gradient in eight radial directions with Gaussian smoothing. This method compares the maps with 12 shapes. In other words, the shapes are considered relevant features. Some recent methods take advantage of recent advances in deep learning. DeepICP [38] was proposed as an approach for a new generation of registration methods based on end-to-end learning. DeepICP solves the problems of ICP registration. BirdNet [39] collects important patterns from a bird’s eye view, which are 2D images of projected LiDAR measurements. Transfer learning, such as the VGG16 architecture, is used for this purpose. Fast-Region-based Convolutional Neural Networks (Fast-RCNN) are also used for object detection. As an extension of this work, BirdNet+ [40] is proposed, which applies an end-to-end strategy directly to 3D point clouds instead of using projection pre-processing. Both methods have been tested on the Kitti object detection benchmark dataset and have shown good results. More information about the registration can be found in [27]. The authors of [41] proposed a method to solve localization and mapping based on the use of a clustering-modified particle filter that selects the best candidate positions using sigma point selection techniques that have the same concept as the unexposed Kalman filter. These points speed up the localization process and also improve accuracy to achieve excellent results. This method also uses non-semantic features to perform the measurement update step in the particle filter. Non-semantic methods are suitable for creating lightweight feature maps, i.e., feature maps can be created in less time because most of these methods do not use complicated calculation formulas. In addition, they can represent any object inside the environment or at least part of them, which makes them robust against any change in the environment.

## 3. Methodology

**Figure 1:**
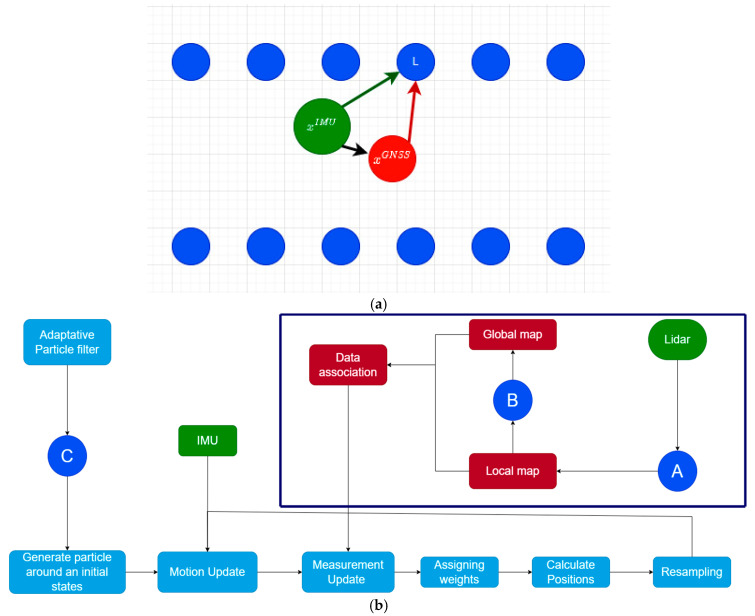
Workflow architecture describing our proposed method. (**a**): Describing the vehicles true position (in red), IMU’s position (in blue) and objects in the vehicles environment as picked up by the LiDAR (in blue). (**b**): The structure of the proposed approach consists of two main steps: data processing {features extraction (A) + global mapping (B)}, described within the dark blue rectangle, which is a framework for LiDAR measurements processing and creating local and global maps. Secondly, localization (C) using adaptive particle filters.

In this section, we present the proposed methodology for solving the problem of localization and mapping in GNSS deprived environment. In Figure 1b, a general overview of the architecture of the proposed approach is shown, where an adaptative particle filter is employed to correct the error provided by the motion and measurement sensor (IMU and LiDAR, respectively) and find the best position estimates. Our method used two kinds of information sources (inputs): LiDAR and IMU.

**The issue:** The IMU unit is responsible for providing information about the vehicle’s movement (translation and rotation). Based on that, the vehicle’s position is estimated by dead reckoning (based on initial position information provided by the GNSS) over defined time intervals. However, the noise present within the IMU’s measurements leads to an exponential error growth which is cascaded over time during the continuous position estimation process. This error can be expressed mathematically as:

$$Err\;\approx x_{t}{}^{IMU}-x_{t}{}^{GNSS}$$

where $x_{t}{}^{GNSS}$ is the real position to estimate (red dot in Figure 1a) provided by the GNSS, and $x_{t}{}^{IMU}$ is the *IMU* derived position of the vehicle (green dot in Figure 1a). Where $x_{t}{}^{IMU}$ is the mathematically derived transformation of the double integrated displacement measurement by the integrated heading rate as described in detail in [6].

**The approach to minimise this error:** In order to overcome this issue, we proposed Adaptive Particle filter which is probabilistic method that takes as an input; the IMU’s information $x_{t}{}^{IMU}$ (containing errors) and a second source of information; the LiDAR’s measurement, which captures objects within the vehicle’s environment and the distances of these objects from the vehicle in the form of 3D points. Using the information of the resolved global position of the object L as illustrated in Figure 1a and the distance between the object L and the vehicle as derived from the LiDAR’s measurement, the IMU’s positional resolving can be corrected.

Our architecture method consists of two main steps: data processing (the dark blue rectangle in Figure 1b) and the localization step. Data processing (feature extraction + mapping) is responsible for extracting relevant features from LiDAR’s measurement and creating local and global maps. In addition, it ensures the fluidity of the matching search between the local and global maps. In the localization phase, the estimation of the vehicle positions is performed using the APF extension. The features extracted, and the IMU information, will feed the input of the APF. Next, some mathematical operations and probabilistic concepts will be applied over the received input (as explained in Section 3.2). Finally, the method provides the estimated positions.

### 3.1. Features Extraction and Local Maps Creation

**Figure 2:**
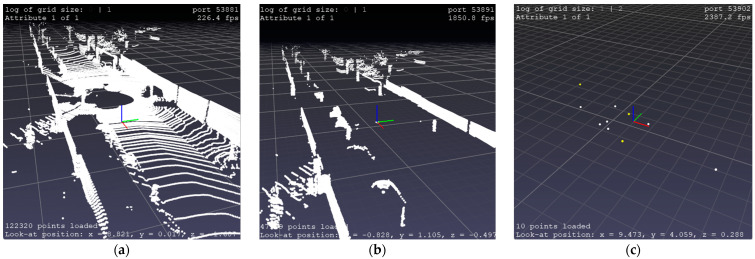
Illustration of the feature extraction. (**a**) 3D LiDAR points scan; (**b**) Removing the ground plan Scene; (**c**) Features extraction process (Zoomed in).

**Figure 3:**
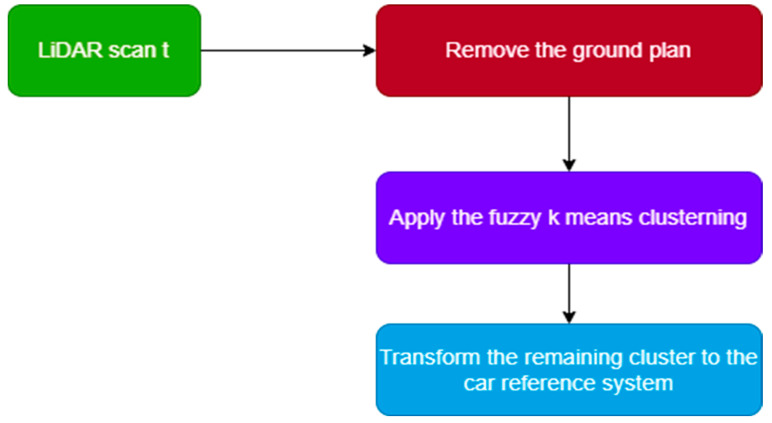
Flowchart of the feature extraction from LiDAR measurement.

The features extraction step, which corresponds to step (A) in Figure 1b, was performed to treat the massive amount of LiDAR data points in order to speed up the localization task and create a lightweight feature map. In this stage, each LiDAR scan, as shown in Figure 2a, is processed as follows. Firstly, we removed the ground plan by excluding points on the *z*-axis of the scan less than 0.1 m, as shown in Figure 2b, as the ground plan does not contain distinguishable features that could help in the matching process and adds to the computational complexity of the approach. Secondly, we applied a Fuzzy K-means clustering to the remaining points and extracted central clusters representing our local feature map, as shown in Figure 2c. In contrast to semantic features, our features are capable of representing less textured environments, such as poles, trees, roads, and curves, without knowing which cluster corresponds to semantic shape features, e.g., poles or trees. We chose to use Fuzzy K-means due to its ability to obtain clusters of different shapes and sizes and reduce the information of a group of points to a central cluster, which is very important for fast interaction within the framework. Moreover, in Table 1, we demonstrate its speed by testing different clustering methods on sequence 0001 from the Kitti data set [30]. A flowchart of the following process is provided in Figure 3, underpinned by an illustration in Figure 2.

Thirdly, we transform all the scans into the vehicle reference coordinate system [21].

### 3.2. Localization

The last stage is the localization part (step (C) in Figure 1b), where we explore the results of the pre-processing steps (features extraction and mapping) to correct the accumulated IMU errors based on an adaptative particle filter described below:

- Particles generation

**Figure 4:**
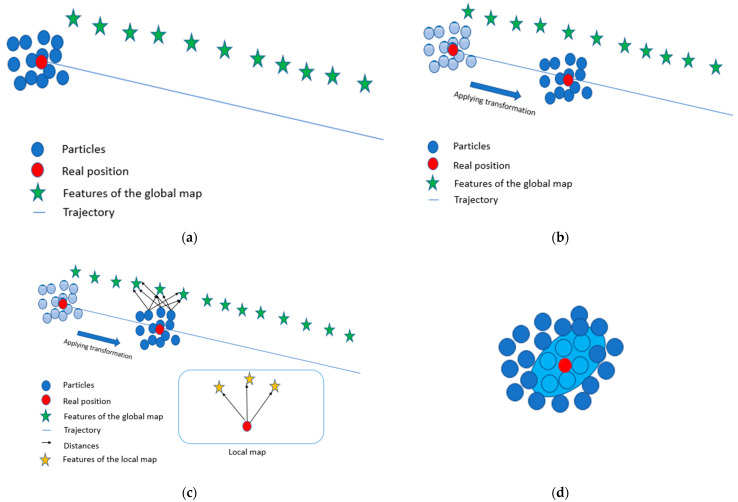
Explanation of the workflow of the particle filter (the images are not real; they are generated to explain the process in depth). (**a**) Generate particles. (**b**) Applying IMU transformation. (**c**) Compare the distances of the particles with the futures of the global map (features in green) and the real distance. (**d**) Resampling stage, where we regenerate particle around the closest one (particle in-cyan color).

The filter generates hundreds of particles around an initial state provided by the GNSS noted by $X={\left\{X_{Par_{i}}\right\}}_{i\in \left[0,N\right]}$ a set of particles where N is the number of particles (see Figure 4a). Each particle is represented as a 4 × 4 homogeneous coordinate matrix resulting after uniformly generating the *x*,*y* coordinates and θ orientation, which is given by:

(3) $$X_{par_{i}}=\left[\begin{array}{cccc} cos\left(\theta_{i}\right) & -sin\left(\theta_{i}\right) & 0 & x_{i}\\ sin\left(\theta_{i}\right) & cos\left(\theta_{i}\right) & 0 & y_{i}\\ 0 & 0 & 1 & 0\\ 0 & 0 & 0 & 1 \end{array}\right]$$

This particular representation of the homogeneous coordinate matrix since there is no rotation about the *x* and *y* axes when the vehicle moves (i.e., the roll and pitch angle are neglected), and the value of the *z* coordinates is neglected because the vehicle is assumed to be driven on a 2D surface. The main advantage of this representation is the simplification of the matrix calculation (i.e., multiplication). Each particle represents a possible target state and is associated with a probability value, called weight $\omega_{par_{i}}$, which is uniformly initialized. The largest of these values belongs to the particle corresponding to the true position of the target.

- Motion update

In the second step of the localization stage using IMU and PF, the motion update moves the particles from scan *t* − 1 (or timestamp *t* − 1) to scan *t* (or timestamp *t*) by using the homogeneous coordinate matrix properties, which perform the transformation with this formulation:

(4) $$X^{t}=\left\{X_{par_{i}}^{t}\right\},X_{par_{i}}^{t}=G^{t}*X_{par_{i}}^{t-1}$$

$G^{t}$ is a 4 × 4 homogeneous matrix of this form:

(5) $$\left[\begin{array}{ccc} . & . & .\\ . & R^{t} & .\\ . & . & .\\ 0 & 0 & 0 \end{array}|\begin{array}{c} .\\ T^{t}\\ .\\ 1 \end{array}\right]$$

$R^{t}$ (3 × 3 matrix) and $T^{t}$ (3 × 1 vector) are the rotation and translation matrices provided by the IMU sensor at scan t, respectively, as shown in Figure 4b.

- Measurement update

The third stage is the measurement update, which examines the information from the measurement sensors, especially the LiDAR sensors, since the information from the camera is sensitive to changes in light and weather conditions. The aim is to determine how similar the transformed (We calculate the distances of each particle with all the features gathered in scan *t*) particle $X_{Par_{i}}^{t}$ provided by the IMU at scan t, is to the real state, i.e., how much it contributes to the determination of the real state. In order to find that, a weight (or percentage) $\omega_{par_{i}}$ is assigned to each particle and updated at each scan, which gives the probability of the particle’s contribution along the trajectory, as shown in Equation (8). According to [21], these weights can be updated by:

(6) $$w_{Par_{i}}^{t}=\underset{p\in \left[0,t\right]}{\prod }P\left(F^{p}|X_{Par_{i}}^{p},M_{n\left(p\right)}\right)$$

where

(7) $$P\left(F^{p}|X_{Par_{i}}^{p},M_{n\left(p\right)}\right):=N\left(∥X_{Par_{i}}^{p}F^{p}-M_{n\left(p\right)}∥,\sigma \right)$$

The product of $X^{p}$ and $F^{p}$ gives the distances between the particles and features at scan p. σ is an isotropic position uncertainty depending on the reference of features. $n\left(p\right)$ is an index of the global features associated with the $p_{th}$ local feature (provided by kd-tree), which means that $M_{n\left(p\right)}$ is the corresponding of $F^{p}$ in the global map M. The idea behind this weight modification is that we can compare the normal curve of distance particle-to-feature (The transformed particles provided by the IMU at scan t) and what we have on the real map (global map). The closer the distributions, the higher the value the weights are (see Figure 4c).

The state estimation at scan t can be calculated by:

(8) $$pos^{t}\approx \sum_{i=1}^{N}w_{Par_{i}}^{t}X_{Par_{i}}^{t}$$

where

(9) $$\sum_{i=1}^{N}w_{Par_{i}}^{t}=1$$

and *N* is the number of particles.

- Resampling

To obtain a fast and accurate method, a resampling step was carried out to reduce the uncertainty of the particles and to adjust their distribution.

By checking the corresponding weights, the particles were ranked from important to unimportant.

The particles were resampled, focusing on the 10 most important particles, which enabled the particles to track the real position at each scan and obtain intuitions about the direction of the positions. We calculated the mean and covariance of the selected particles. Then, we regenerated the particles based on the multinormal distribution (see Figure 4d).

- Evaluation

Our metric evaluation included six error measurements $\Delta_{pos}$, $\Delta_{lat}$, $\Delta_{lon},\wedge \Delta_{ang}$ denoting the mean absolute positional, latitudinal, longitudinal, and heading errors, respectively, while $RMSE_{pos}$ and $RMSE_{ang}$ represent the corresponding Root Mean Squared Errors of the position and heading estimation.

(10) $$\begin{array}{c} \Delta_{lat}=\frac{\sum_{i=1}^{n}\left|x_{i}-x_{i}^{\prime }\right|}{n},\Delta_{lon}=\frac{\sum_{i=1}^{n}\left|y_{i}-y_{i}^{\prime }\right|}{n}\\ \Delta_{pos}=\frac{\sum_{i=1}^{n}∥pos_{i}-pos_{i}^{\prime }∥{}_{2}}{n},\Delta_{ang}=\frac{\sum_{i=1}^{n}\left|\theta_{i}-\theta_{i}^{\prime }\right|}{n}\\ RMSE_{pos}=\sqrt{\frac{\sum_{i=1}^{n}∥pos_{i}-pos_{i}^{\prime }∥{}_{2}^{2}}{n}},RMSE_{ang}=\sqrt{\frac{\sum_{i=1}^{n}{\left|\theta_{i}-\theta_{i}^{\prime }\right|}^{2}}{n}} \end{array}$$

where *n* is the scan number, $pos_{i}=\left[x_{i},y_{i}\right]$ is the predicted state, ${pos}_{i}^{\prime }=\left[x_{i}^{\prime },y_{i}^{\prime }\right]$ is the actual state, $x_{i}$ and $x_{i}^{\prime }$ are the predicted and actual lateral, $y_{i}$ and $y_{i}^{\prime }$ is the predicted and actual longitudinal. $\theta_{i}$ is the predicted angle, and $\theta_{i}^{\prime }$ is the actual angle.

We used the longitudinal and latitudinal mean absolute error $\Delta_{lat}$ and $\Delta_{lon},$ respectively, to examine the causes of error fluctuation according to the *x*-axis and *y*-axis, such as hard braking, vehicle rotation, or vehicle acceleration. We used the mean absolute position and root mean square positioning error $\Delta_{\mathrm{pos}}$, $RMSE_{pos}$ to study the overall localization error and the effect of the vehicle rotation error $\Delta_{ang}$, $RMSE_{ang}$ on that error.

## 4. Results and Discussion

### 4.1. Experiments

A computer with an 8th generation i7 processor and 20 GB of RAM was used in our experiments. We chose the Kitti dataset to test the proposed method on several sequences characterizing urban, residential, campus, and pedestrian traffic. The advantage of using this dataset is that it provides measurement information from a variety of sensors, including LiDAR point clouds, stereo or mono camera images, IMU inertial measurements, and high-accuracy GNSS data. Kitti also enables the evaluation of proposed approaches in various environments. We tested our method on a second dataset, the Panda dataset [42], which contains different driving scenarios, including steep slopes, construction sites, heavy traffic, and pedestrians, as well as a variety of weather and lighting conditions, such as in the morning, afternoon, evening and night. The dataset is equipped with various sensors, such as IMU/GPS information and Lidar, for camera measurements. A 10-core feature extraction method in the K-means algorithm was used to create a global map (or a ground plan) based on a high-accuracy navigation system. Two criteria were considered in selecting the number of clusters: speed and representativeness. Smaller clusters are faster but less representative, and vice versa. Each local map was matched with a global map, and the localization process was started using particle filters. The number of particles was around 100 points. The initialization region was also in the range [−i, i], where i is a random number within the range [0, 1]. The output of the localization algorithm is a 4 × 4 information (transformation) matrix containing the matrices of rotation and translation performed between two consecutive keyframes. The method was tested on the entire class of Kitti datasets, which contained a total of 987 scans from four sequences. The codes used in this experiment can be found in [43].

**Figure 5:**
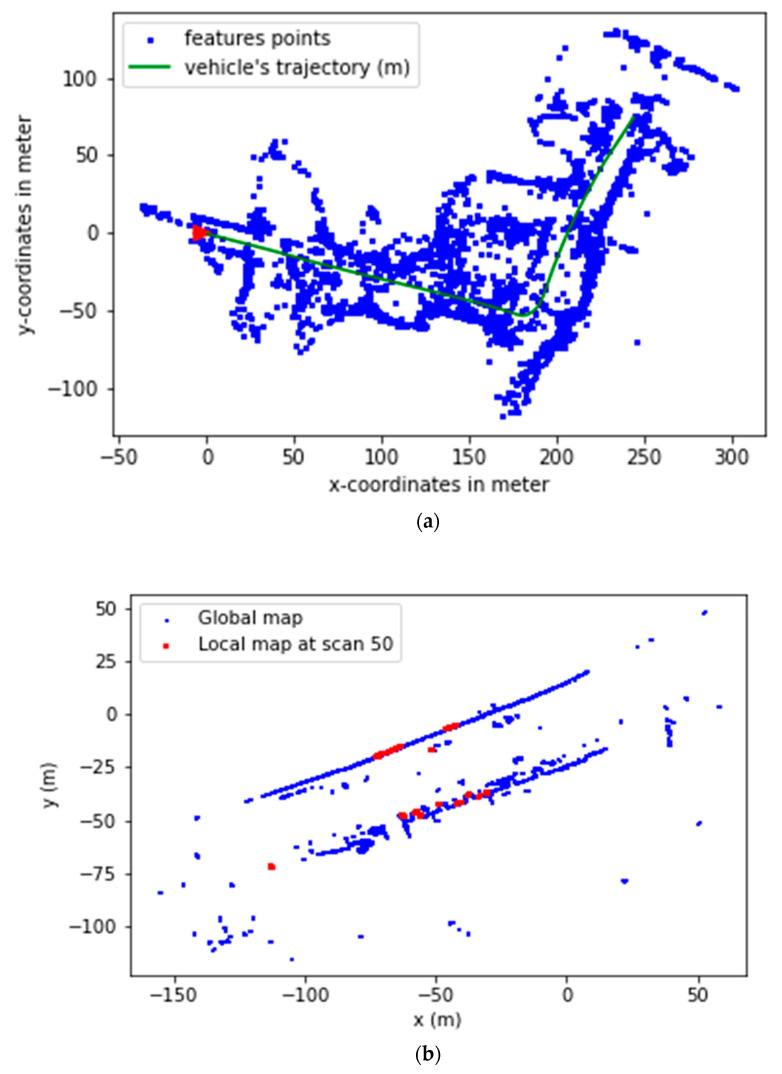
(**a**) The results of the mapping phase after concatenation and transformation. The blue dots are the global features map, the red triangle is the particle initialization, and the green line is the ground truth. (**b**) Example of data association of scan 50 of the sequence 0001 of the Kitti dataset.

For the purpose of this experiment, the global maps were created priorly using the LiDAR scans. After creating the local maps and converting them into a reference system., we merge them in an order where each time frame corresponds to a single local map, which creates a global map of the trajectory. Creating a global map (step (B) in Figure 1) is an essential step before executing any localization method. As mentioned before, it acts as a guide for the vehicle, i.e., it provides distances to the objects. This kind of map is a low-content information one, meaning that it represents objects (or part of them) by providing only their coordinates (see the illustration in Figure 5a). The creation of the global map with this technique speeds up the process of data association. Data association is the task of finding a similar part of the local map in the global map by using the kd-tree searching algorithm. Example of this process can be seen in Figure 5b.

Let us note:

(11) $$k\in \left[0,M_{f}\right],M=\left\{m_{k}\right\}$$

where $M_{f}$ is the number of feature points in the global map, $m_{k}$ is the feature point at position *k*, and *M* is the list of features in the global map. Let us also define:

(12) $$k\in \left[0,N_{f}\right],F^{t}=\left\{f_{k}^{t}\right\}$$

with $N_{f}$ the number of feature points in the LiDAR scan *t*, $f_{k}^{t}$ the feature point at position *k* in the scan *t*, and $F^{t}$ the list of features at scan *t*. It is important to note that the use of LiDAR scans in our experiments doesn’t represent map matching in real world scenarios.

### 4.2. Discussion

- Parameters discussion:

We have investigated the parameters of a configuration of our features extraction framework in terms of fastness and accuracy in order to find the optimal parameters that give the best results. The most important parameter that should be investigated in the features extraction workflow is the number of clusters in the fuzzy K-means algorithm.

**Figure 6:**
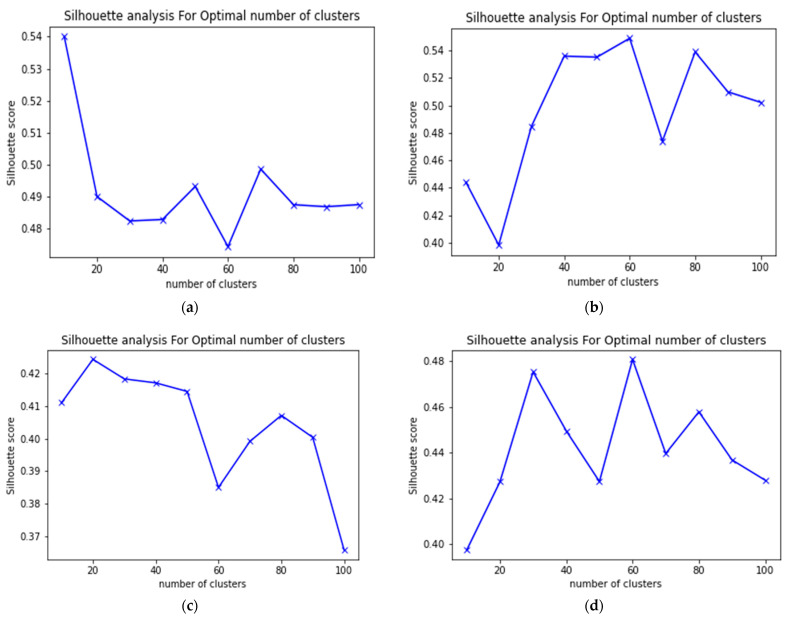
Silhouette values registered by fuzzy K-means algorithm for different cluster numbers in sequences 0001, 0034, 0027, and 0053 from the Kitti dataset. (**a**) Silhouette values in sequence 0001. (**b**) Silhouette values in sequence 0034. (**c**) Silhouette values in sequence 0027. (**d**) Silhouette values in sequence 0053.

We calculated the silhouette values for different numbers of clusters in different sequences of the Kitti dataset. We observe in Figure 6 that the average silhouette value for all sequences is 0.44 with a cluster number of 10, 0.43 with a cluster number of 20, 0.46 with a cluster number of 30, 0.47 with a cluster number of 40, 0.46 with a cluster number of 50, 0.46 with a cluster number of 60, 0.45 with a cluster number of 70, 0.47 with a cluster number of 80, 0.45 with a cluster number of 90, and 0.44 with a cluster number of 100. We saw that the silhouette value of all scenarios is in the range of 0.43–0.47, which justifies the choice of a 10-core clustering center, as it consumes less time and regularly has a good silhouette value.

Our feature extraction process, which is illustrated in Figure 2, effectively reduces the time and cost of calculation, where each scan of size 1.8 MB (on average) was processed in 0.4 s (on average) and reduced into 790 KB (on average), which demonstrates the lightweight of our global and local maps. Moreover, the process helps the data association stage to be fast, facilitating investigations on similarity issues.

- Discussion on Kitti and Pandaset accuracies:

The results in Table 2 show that the method can reliably locate the vehicle in various sequences and categories, such as weather and seasonal changes. In addition, the method takes 7 s to locate the vehicle within 5 sequences and 544 frames, which is fast enough to operate in a real-time scenario; the IMU sensor takes 52 s to record information from 544 frames, and our method quickly processes the incoming data with an execution time of 7 s. The manual testing of the Schaeffer et al. [21] method on the sequence 0001 (City category) of the Kitti dataset showed an absolute position error of 0.06 m and an absolute rotation error of $0.1^{{}^{\circ}}$ in 19 s. In our case, we obtained 0.07 m for the absolute position error and $0.013^{{}^{\circ}}$ for the rotation error in a run time of 6 s, as presented in Table 2, which demonstrates the fastness of our method. According to Table 2, we registered 0.25 m of the average positioning error of all the sequences in only 7 s of execution, which demonstrate the capability of our method to localize the vehicle with a minimum of 100 particles. The obtained results are due to the robust resampling technique that followed accurately the trajectory.

In another part, we tested the accuracy of our method against four methods that we found relevant in our state-of-art survey: Kümmerle et al. [19], Weng et al. [20], Sefati et al. [18], and Schaefer et al. [31]. All of them have used semantic features to represent LiDAR scans, such as poles, walls, trees, etc. The comparison of Sefati et al. [18] and Schaefer et al. [21] approaches with our proposed method was feasible, as they used the same error metrics, and their implementation was provided in their paper. However, it was hard to compare our method with Weng et al. [20] and Kümmerle et al. [19] method unless qualitatively, as it was provided by A. Schaefer et al. in the article [21]. Table 3 shows that our method can produce competitive results. In fact, our method outperformed all others in terms of mean absolute angular error and mean square angular error. In addition, we obtained competitive results for mean absolute positioning error and mean squared positioning error, which outperformed all compared methods except Sheafer et al. [21], (with our results 0.09 m and 0.11 m, respectively) and Sheafer et al. [21] (0.11 m and 0.12 m, respectively, for our method).

These results are justified by the robust data association in the work of Sheafer et al. [21]. However, working with semantic features can affect the localization process, especially when these features are not present in the environment.

We extensively tested our method on a second dataset, Pandaset, on 5 sequences containing 80 frames each. Our method recorded an average absolute positioning error of 0.16 m in 5 s runtime, demonstrating the performance of our method when dealing with different environmental scenarios (see Table 4).

**Figure 7:**
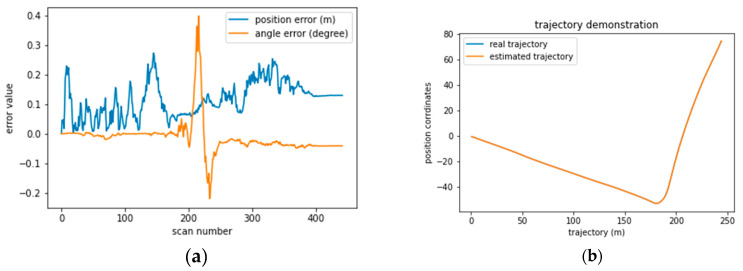

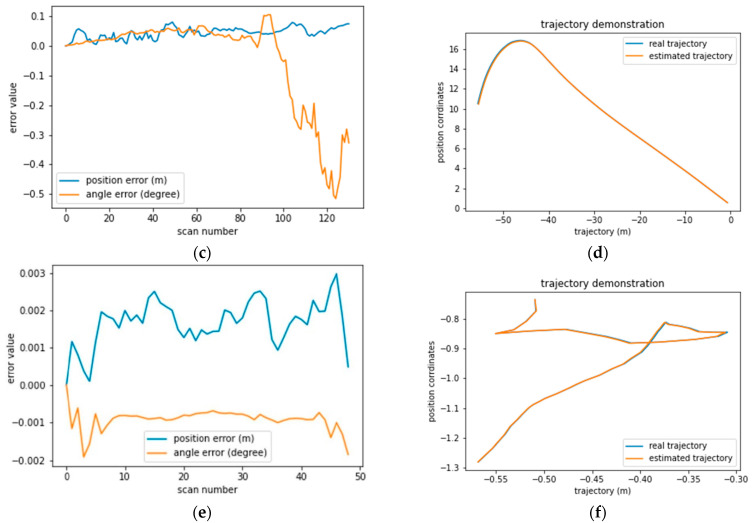
Graphical display of the average absolute position and angular error relative to the vehicle path for each sequence. (**a**) positional and angular error in seq 0009. (**b**) vehicle trajectory in seq 0009. (**c**) positional and angular error in seq 0035. (**d**) vehicle trajectory in seq 0035. (**e**) positional and angular error in seq 0034. (**f**) vehicle trajectory in seq 0034.

We studied in depth the results of sequence 0009. In Figure 7a,b, we mapped the position and angular error variations in each scan, allowing us to make some relevant observations. We found that acceleration mainly affects the accuracy of positioning. From scan 0 to 20, the period in which the vehicle takes off, and from scan 110 to 170, the period in which the vehicle slows down when taking a turn on the road (Figure 7b). In both periods, the error varies from 0 m to 0.25 m and back to 0 m, which means that the particles need time to adapt to the new acceleration of the vehicle.

**Figure 8:**
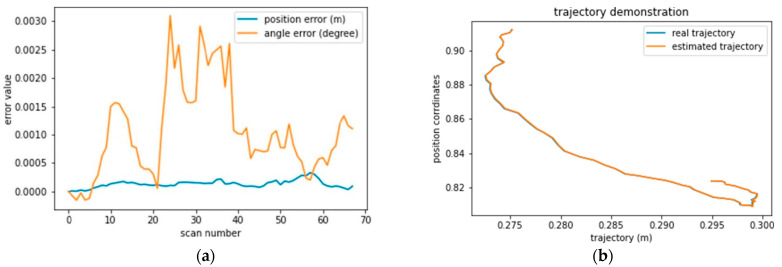

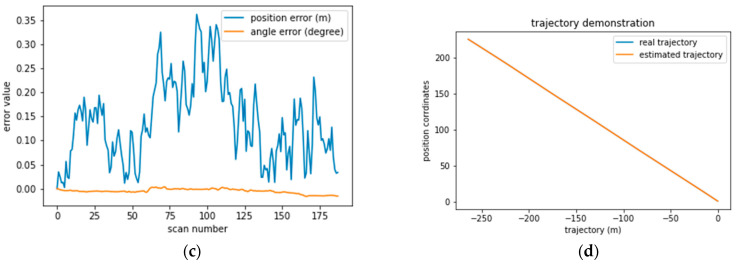
Graphical display of the average absolute position and angular error relative to the vehicle path for each sequence. (**a**) positional and angular error in seq 0053. (**b**) vehicle trajectory in seq 0053. (**c**) positional and angular error in seq 0027. (**d**) vehicle trajectory in seq 0027.

The same is observed in the case of drift or hard breaking, like in Figure 7c,d and Figure 8c,d sequences 0035 and 0027. Therefore, a good positioning model should take into account the velocity to anticipate the vehicle’s motion. Furthermore, we observe that the positioning error is systematically 0.1 m from scan 170 to 250 when the vehicle is turning. However, the angular error recorded high values from 0 to 0.4, indicating the need to determine the turning intensity. Additionally, the same issue was registered in sequence 0034 in Figure 7 and sequence 0053 in Figure 8.

As research in the field of localization and mapping shifts from observational studies to direct applications in real-world scenarios, the need for methods that speed up the localization process, such as the method we propose in this paper, is increasing. However, there are some drawbacks to the use of particle filtering algorithms, particularly in setting the particle number and threshold identification.

## 5. Conclusions and Future Work

This paper presents a method for autonomous vehicle positioning and mapping based on non-semantic feature extraction. A fuzzy K-means clustering was used to extract features from LiDAR scans. The cluster centroids features were used to create local map features. Furthermore, an adaptive particle filter was used in the localization process, which included a resampling stage after finishing the measurement updates in order to increase the reliability of the position estimation and reduce the time and energy cost. The resampling method selects the closest 10 particles to the real position by checking their weights (percentage) and regenerating particles around them using a multinormal distribution.

The proposed method provides competitive accuracy results in significantly less time compared to the state-of-the-art methods evaluated on the Kitti database. We obtained an error of 0.25 m in the mean position error of all sequences in the Kitti dataset in a run time of 7 s. Furthermore, we obtained 0.15 m in the mean position error of all sequences in the Pandaset dataset in only 5 s of execution, which demonstrates the potential of our resampling contribution to speed up the localization process and obtain state-of-the-art results. The proposed method provides competitive accuracy results in a significantly shorter time compared to state-of-the-art methods that have been evaluated on the Kitti dataset.

However, the accuracy of the proposed method sometimes deviated due to poor initialization of particle filters or poor selection of clusters or number of particles. Therefore, future research will involve finding the optimal initialization of the particle filter and finding the best parameters configuration. The particle filter suffers from the random initialization of the particles, which could be modelled by a stochastic differential equation to control particle generation. On the other hand, the use of other clustering methods opens up a new approach to the representation of Lidar measurements, which can provide insights into the use of other machine learning techniques, such as object tracking with deep learning (or transfer learning), to ensure better matching of data association. Thus, the position of the vehicle could be more accurately estimated.

## Figures and Tables

**Table 1:** Time consumed (mm:ss) in executing the feature extraction workflow with the specified clustering methods tested on the sequence 0001 from the Kitti data set [30].

Clustering Method	Time Cost
Growing neural gas	01:24
KMeans	01:38
Fuzzy K-means	00:43
Hierarchical clustering	01:07
Gaussian mixture model	00:49
Self-organizing maps	02:06
Agglomerative clustering	01:18
Particle swarm optimized clustering	00:52

**Table 2:** Kitti localization error obtained in different categories and the time consumption in each sequence.

Category	Seq	frames no	*tFeat* (s)	*tloc* (s)	∆*_pos_* (m)	∆*_lat_* (m)	∆*_lon_* (m)	∆*_ang_* (°)	*RMSE_pos_* (m)	*RMSE_ang_* (°)
City	0001	108	00:34	00:01	0.07	0.01	0.06	0.012	0.08	0.014
Residence	0035	131	00:43	00:02	0.038	0.019	0.02	0.09	0.053	0.18
Road	0027	188	01:08	00:02	0.15	0.03	0.14	0.01	0.17	0.006
Campus	0034	49	00:16	00:01	0.0009	0.0006	0.0005	0.0014	0.001	0.001
Person	0053	68	00:23	00:01	0.0001	0.0001	0.0001	0.001	0.0007	0.0002

tfeat: the cost of time to calculate the features extraction. tloc: the cost of time to calculate the localization.

**Table 3:** Performance comparison of the proposed technique. The results of Weng et al. [20] and Kümmerle et al. [19] are not straightforwardly similar and are expressed for qualitative analysis only.

Methods	∆*_pos_*(m)	*RMSE_pos_* (m)	∆*_lat_*(m)	$\sigma_{lat}$ (m)	∆*_lon_*(m)	$\sigma_{lon}$ (m)	∆*_ang_*(°)	$\sigma_{ang}$ (°)	*RMSE_ang_*(°)
Kümmerle et al. [19]	0.12	—	0.07	—	0.08	—	0.33	—	—
Weng et al. [20]	—	—	—	0.082	—	0.164	—	0.329	—
Sefati et al. [18]	—	0.24	—	—	—	—	—	—	0.68
A. Schaefer et al. [21]	0.096	0.111	0.061	0.075	0.060	0.067	0.133	0.188	0.214
Charroud. A et al. [28]	0.12	0.141	0.059	0.09	0.08	0.05	0.043	0.078	0.057
Ours	0.101	0.12	0.064	0.035	0.06	0.087	0.043	0.075	0.075

**Table 4:** Accuracy evaluation of our method in the Pandaset dataset.

Seq	frames no	*tFeat* (s)	*tloc* (s)	∆*_pos_* (m)	∆*_lat_* (m)	∆*_lon_* (m)	*RMSE_pos_* (m)
100	80	00:01	00:01	0.18	0.07	0.16	0.21
109	80	00:01	00:01	0.22	0.07	0.20	0.23
117	80	00:03	00:01	0.16	0.06	0.13	0.19
139	80	00:01	00:01	0.22	0.03	0.21	0.29
158	80	00:01	00:01	0.05	0.03	0.03	0.06

## Data Availability

In this article, the Kitti dataset was used [29], which is available for free download.
